# First report on natural infection by *Dirofilaria repens* in a cat in Spain: case report and literature review of feline subcutaneous dirofilariosis in Europe

**DOI:** 10.1007/s11259-023-10250-7

**Published:** 2023-11-03

**Authors:** Sergio Villanueva-Saz, María Victoria Martínez, Sandra Alsina, Antonio Fernández, Álex Gómez, Maite Verde, Héctor Ruiz, Delia Lacasta, Alaa Aldin Alnassan, Michele Trotta, Andrés Yzuel, Diana Marteles

**Affiliations:** 1 Clinical Immunology Laboratory, Veterinary Faculty, University of Zaragoza, Zaragoza, 50013 Spain; 2 Department of Animal Pathology, Veterinary Faculty, University of Zaragoza, Zaragoza, Spain; 3 Instituto Agroalimentario de Aragón-IA2 (Universidad de Zaragoza-CITA), Zaragoza, Spain; 4 Centro Clínico Veterinario Teruel, Teruel, Spain; 5 Clinica Veterinaria Bitxets, Grao de Castellón, Spain; 6 Department of Parasitology, Veterinary medicine laboratory IDEXX, Kornwestheim, Germany

**Keywords:** Cat, *Dirofilaria repens*, Europe, PCR, Spain

## Abstract

*Dirofilaria repens* is an endemic mosquito-borne pathogen widely spread throughout Europe and other non-Europen regions. Infection by *D. repens* has been reported in dogs, although little is known about the occurrence and epidemiological features of this nematode in cats. During the surgical procedure within the framework of a trap, neuter and release sterilization program, two female filarial nematodes with spontaneous movement were removed from the internal part of the spermatic cord from asymptomatic stray cat in Spain (Grao de Castellón, Castellon de la Plana, province of Spain). Moreover, the presence of microfilariae was detected by using three different methods, including direct blood smear technique, the microhematocrit tube test, and Knott´s modified test. PCR performed from the nematodes extracted from the spermatic cord and from microfilariae in EDTA-blood sample tested both positive for *D. repens*. To the authors’ knowledge, this is the first report that demonstrates the presence of *D. repens* in cats in Spain. In this sense, a review of the current scientific status of feline subcutaneous dirofilariosis in Europe was also performed. Future investigations should analyze the epidemiological role of cats in *D. repens* infection including the prevalence of infection. Veterinarians working in endemic areas should be aware of this infection in cats and their susceptibility.

**Supplementary Information:**

The online version contains supplementary material available at 10.1007/s11259-023-10250-7.

## Introduction

Subcutaneous dirofilariosis is a zoonotic disease caused by *Dirofilaria repens*, a vector-borne filarial parasite transmitted by the bite of infected competent mosquito species. In Europe, there are different mosquito species implicated in the transmission of *D. repens*, the larva migrates to the mosquito’s proboscis, and when it takes a blood meal it releases the infective third-stage larvae (L3) along with the saliva and the larvae actively penetrate through the orifice into the host (ESDA 2017). Over time, the infecting stage develop into adult parasites, which can colonise the subcutaneous tissues in various parts of the body. Other animals can be infected including cats with the description of clinical cases; however, the epidemiological role of this species is minimal as the cat is considered an imperfect host (ESDA 2017).

This study describes for the first time the diagnosis and isolation of subcutaneous dirofilariosis caused by *D. repens* in a naturally infected cat in an endemic region of Spain; a review of the current scientific status of feline subcutaneous dirofilariosis in Europe was also performed.

## Materials and methods

### Case history

**Fig. 1:**
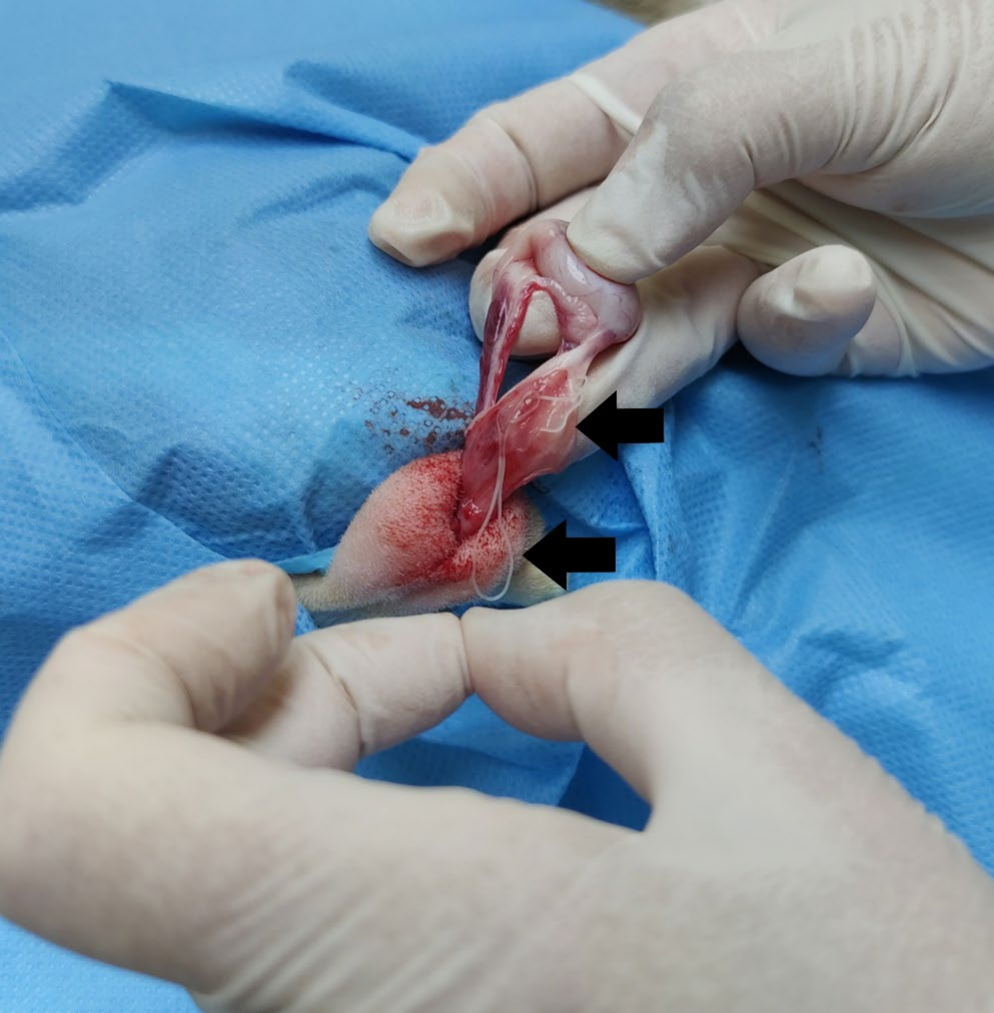
Presence of the filarial nematodes in the internal part of the spermatic cord (arrows)

The case was an adult (> 1 year old) intact male cat captured in July 2023 in the urban area of Grao de Castellón (39º 58′ 00′′ N, 10º 10′ 00′′ W, Castellon de la Plana, province of Spain) within the framework of a trap, neuter and release sterilization program run locally to control stray cat populations. During the surgical procedure, two female filarial nematodes with spontaneous movement were removed from the internal part of the spermatic cord (Fig. 1).

### Laboratory data collection for hematologic and clinical biochemistry analysis

Three millilitres of blood sample was collected for complete blood count (CBC) and clinical biochemistry, to determine the following parameters: glucose, total protein concentrations, albumin, blood urea nitrogen, creatinine, calcium, inorganic phosphorus, alanine aminotransferase (ALT), aspartate aminotransferase (AST), alkaline phosphatase, gamma glutamyl transferase (GGT), total bilirubin, amylase, globulins, and serum amyloid A. These samples were stored at 4ºC for a maximum of 12 h. Serum protein was also performed by agarose gel electrophoresis system. Laboratory parameters were considered altered when they were outside the reference intervals.

### Diagnostic imaging tests

Thoracic radiography in left lateral view and echocardiography in right parasternal short axis view were also performed in the cat to rule out the presence of adult heartworms or signs consistent with feline heartworm disease.

### Complementary serological and molecular test to evaluate the presence of co-infections

Different co-infections causing immunosuppressive status and/or chronic disease were tested by serology including *Toxoplasma gondii*, *Leishmania infantum*, feline calicivirus (FCV), feline herpesvirus type 1 (FHV-1), feline leukemia virus (FeLV), feline immunodeficiency virus (FIV) and SARS-CoV-2. In the case of SARS-CoV-2 seropositivity in cats, there is evidence that seropositive cats to SARS-CoV-2 infection might be especially susceptible due to existence of concomitant infections with immunosuppressed feline pathogens (Villanueva-Saz et al. 2022). Moreover, the detection of genomic DNA of *Mycoplasma haemofelis*, *Mycoplasma haemominutum* and *Mycoplasma turicensis* was also attempted.

### Detection of the presence of microfilariae in fresh blood samples

A direct blood smear technique, the microhematocrit tube test (MCT), and Knott´s modified test were performed to detect the presence of microfilariae in fresh blood samples. Additional, stained blood films was also performed using Diff-Quik Staining (Liotta et al. 2013).

### Detection of circulating Dirofilaria immitis antigens by rapid test and by Enzyme-linked immunosorbent assay (*ELISA*) technique, and detection of D. immitis antibodies by in-house ELISA technique

The presence of circulating *D. immitis* antigens was investigated by using two different commercial tests, including a rapid test by SNAP Pro Analyzer (IDEXX laboratories, Westbrook, ME, USA), and an ELISA kit (DiroCheck® Heartworm Antigen Test Kit, Zoetis, Florham Park, USA). Moreover, anti-*D. immitis* antibodies were investigated by an in-house ELISA technique based on *D. immitis* pepsin inhibitor Dit33 recombinant protein (Villanueva-Saz et al. 2021).

### Molecular characterization of the adult parasites and EDTA-blood sample

The molecular characterization of filarial nematodes and the EDTA-blood sample was performed by sending samples to the IDEXX laboratories (Barcelona, Spain) to perform real-time polymerase chain reaction (rt-PCR) for panfilarial-4-species detection including *D. repens*, *D. immitis*, *Acanthocheilonema reconditum* and *Acanthocheilonema dracunculoides.*

### Morphological examination of the adult parasites

The parasites were placed in physiological solution and showed spontaneous movement, and were fixed in 70% alcohol for microscopic examination.

### Search strategy and eligibility criteria

A bibliographic search was performed on the PubMed electronic database using the following MeSH search terms: “*Dirofilaria repens*”, “cat”, and “Europe”. A combination of keywords was used: *Dirofilaria repens* AND cat AND Europe. The search was restricted to articles and abstracts from international congress published in English and with date of publication between January 1, 1990, and July 31, 2023. Reference lists of the relevant articles were also screened to identify additional studies. We excluded studies investigating animals different from cats. Special attention was paid to the articles with clinically relevant information.

## Results

### Case clinical observation and clinicopathological findings

A first initial physical examination, showed that the cat had a body condition of 3/5, was normothermic with a rectal temperature of 38.5 ºC, properly hydrated, with pink mucous membranes. Abdominal palpation did not reveal any abnormalities, there was absence of organomegaly or the presence of palpable masses and no abdominal pain. Cardiac auscultation was within normal limits. Respiratory sounds were also normal and there was no evidence of lymph node enlargement. The general clinical examination was unremarkable, and the cat was classified as apparently healthy. The electrophoresis detected an increase in the gamma fraction classified as polyclonal gammopathy. All the laboratory findings about the case are detailed in Table 1.

**Table 1:** Haematological, biochemical parameters determined in the cat

Parameter	Value	Reference range
Haematology		
WBC (K/µl)	15.25	2.87–17.02
Neutrophils (K/µl)	**11.37**	2.30-10.29
Lymphocytes (K/µl)	2.00	0.92–6.88
Monocytes (K/µl)	0.61	0.05–0.67
Eosinophils (K/µl)	0.76	0.17–1.57
Basophils (K/µl)	**0.51**	0.01–0.26
RBC (M/µl)	6.93	6.54–12.20
Haematocrit (%)	31.0	30.30–52.30
Haemoglobin (g/dl)	**9.3**	9.80–16.20
MCV (fl.)	44.7	35.90–53.10
MCH (pg)	13.4	11.80–17.30
MCHC (g/dl)	30.0	28.10–35.80
RDW (%)	23.60	15.00–27.00
Plateles (K/µl)	368	151–600
Reticulocytes (K/µl)	9.7	3.00–50.00
% Reticulocytes	0.1	
Reticulocyte haemoglobin (pg)	16.1	13.20–20.80
Blood chemistry		
Glucose (mg/dl)	155	63–162
Blood urea nitrogen (mg/dl)	18	6–36
Creatinine (mg/dl)	0.8	0.80–1.60
Calcium (mg/dl)	10.2	8.50–11.60
Inorganic phosphorus (mg/dl)	6.2	3.20–8.70
Alanine aminotransferase (U/l)	50	10–85
Aspartate aminotransferase (U/l)	35	10–85
Alkaline phosphatase (U/l)	4	0-110
Gamma glutamyl transferasa (U/l)	9	1–10
Total bilirrubin (mg/dl)	0.1	0.00-0.20
Amylase (U/l)	1275	700–2000
Acute phase protein		
Serum Amyloid A (µg/ml)	**37.3**	5.00–10.00
Electrophoretogram of serum proteins		
Total protein (g/dl)	**8.4**	5.40-8.00
Albumin (g/dl)	3.3	2.10-4.00
Globulins (g/dl)	**5.6**	2.90–4.70
Alpha 1 globulins (g/dl)	0.5	0.10–1.10
Alpha 2 globulins (g/dl)	0.9	0.40–0.90
Beta globulins (g/dl)	**0.7**	0.90–1.90
Gamma globulins (g/dl)	**3.0**	1.30–2.20
Albumin:globulin ratio	0.65	0.45–1.30

Note: Abnormalities are highlighted in bold

Abbreviations: MCH, mean corpuscular haemoglobin; MCHC, mean corpuscular haemoglobin concentration; MCV, mean corpuscular volume; RBC, red blood count; RDW, red blood cell distribution; WBC, white blood count

Thoracic radiography was normal with the absence of vascular enlargement, pulmonary parenchymal inflammation or oedema. In the case of echocardiography, no worms were seen in the lumen of the pulmonary or in the right-side chambers.

### Complementary serological and molecular results to evaluate the presence of co-infections

All serological tests performed to detect the presence of specific antibodies against *T. gondii*, *L. infantum*, FCV, FHV-1, FeLV, FIV and SARS-CoV-2 were negative. Moreover, the presence of DNA of hemotropic *Mycoplasmas* was not detected.

### Detection of the presence of microfilariae in fresh blood samples

**Fig. 2:**
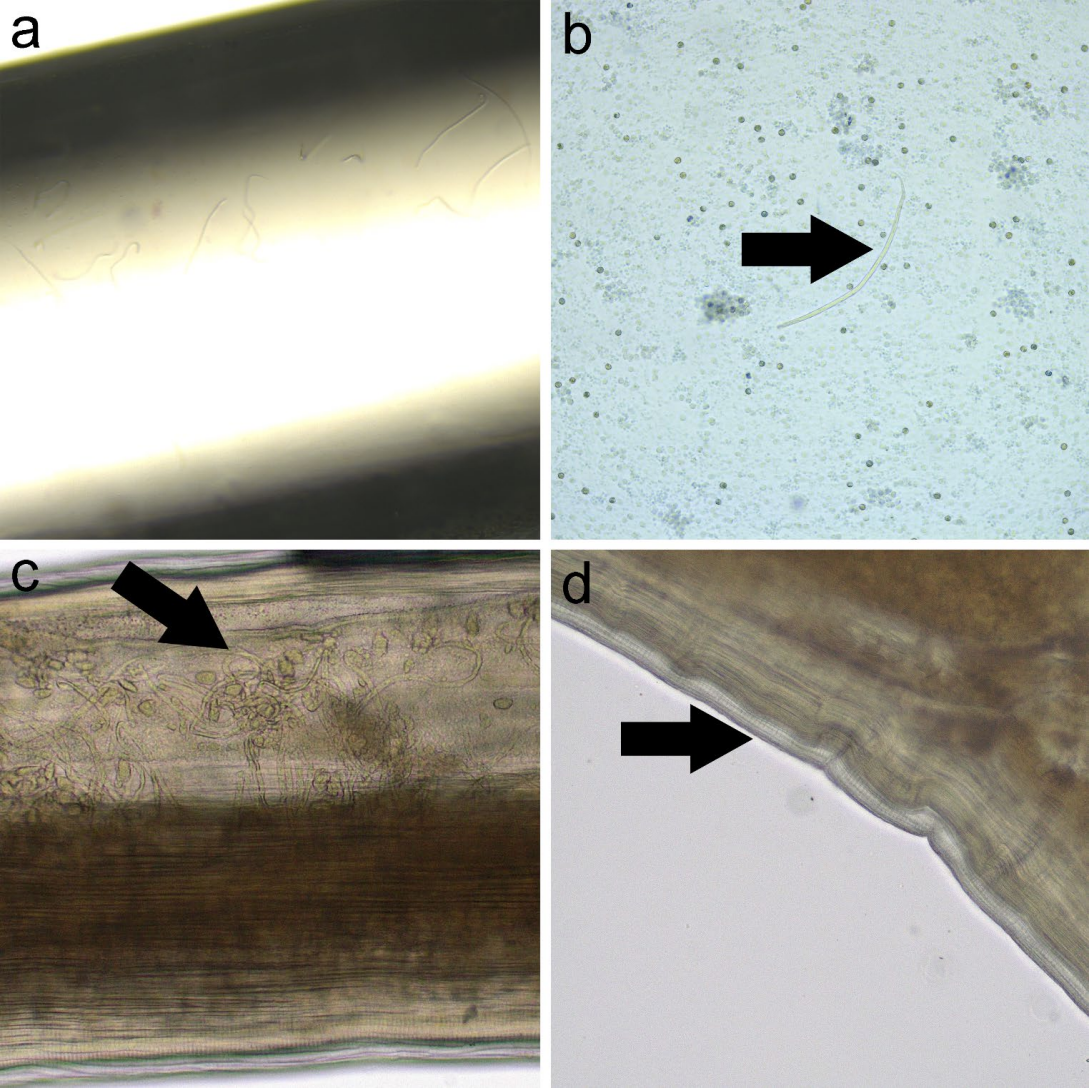
Parasitological examination using different techniques. a). Positive MCT with presence of multiple microfilariae (4x); b). Knott’s test revealed the presence of microfilariae of *D. repens* with the following morphological features: unsheathed with conical cephalic end and the tail like an umbrella’s handle (10x); c). Uterus with microfilaria from adult nematode (100x); d) Cuticular surface with longitudinal ridge from adult nematode (200x)

**Fig. 3:**
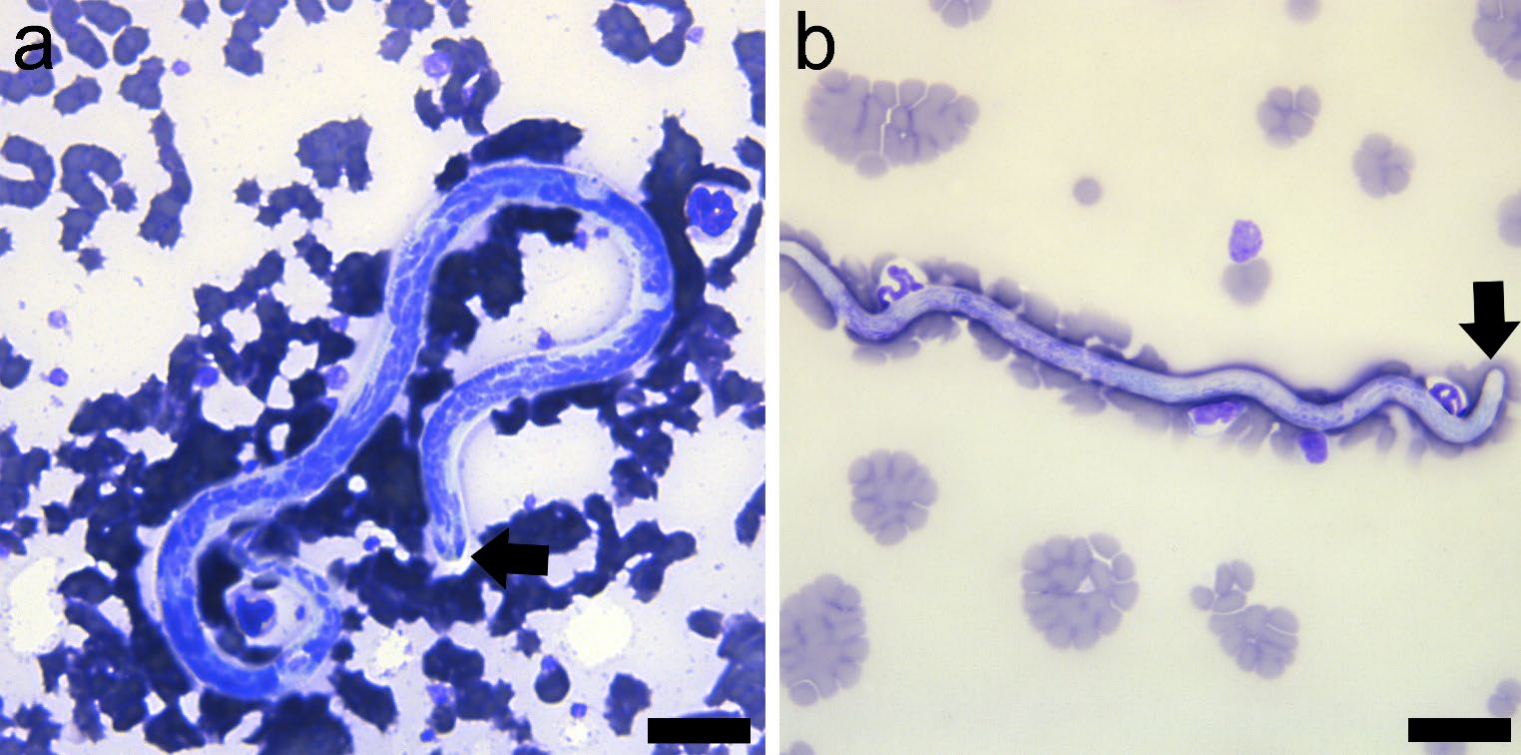
Stained blood films. a). *D. repens* microfilariae with the presence of a pair of nuclei in the cephalic space (arrow) (Diff Quick, bar = 200 μm); b). *D. immitis* microfilarie from a canine clinical case with the absence of a pair of nuclei in the cephalic space (arrow) (Diff Quick, bar = 200 μm)

The presence of microfilariae was detected by using three methods (Fig. 2a and b). The modified Knott’s test revealed the presence of microfilariae of *D. repens* with the following morphological features: unsheathed with conical cephalic end and the tail like an umbrella’s handle (Fig. 2b). Differentiation of the microfilariae of *D. immitis and D. repens* was achieved in stained blood films as previously described by Liotta et al. (2013). In our case, cephalic space of the microfilariae was compatible with *D. repens* because a pair of nuclei was present and separated from the rest of the somatic nuclei of the microfilariae (Fig. 3a) in contrast to *D. immitis* (Fig. 3b).

### Results of detection of circulating D. immitis antigens by rapid test and ELISA and detection of D. immitis antibodies by in-house ELISA

No circulating *D. immitis* antigens and anti-*D. immitis* antibodies were detected with the serological techniques included for this purpose. The results obtained suggested that *D. immitis* was not the filarial parasite involved in the present case report.

### Results of molecular characterization by rt-PCR of panfilarial-4-species of the nematodes and microfilariae

PCR performed from nematodes extracted from the spermatic cord and from microfilariae in EDTA-blood sample tested both positive for *D. repens*.

### Results of parasitological examination of adult parasites

Microscopic features were compatible with *D. repens* morphology. In this sense, the two adult females were composed of a thick cuticle with spaced longitudinal ridges on the surface, large lateral chords, polymyariancoelomyarian musculature, one small intestine, and paired uteri containing morulae and microfilariae (Fig. 2c and d).

### Results of the bibliographic research

A total of 20 articles were identified through data base (Pubmed) searching, and additional eight articles were identified through reference list screening. However, nine articles identified through Pubmed were excluded for the following reasons: heartworm disease in animals (n = 3), subcutaneous dirofilariasis in human (n = 5) and finally an article related to feline subcutaneous dirofilariosis performed from Thailand (n = 1). Finally, 19 articles were included in this review and these selected articles were classified as clinical reports (n = 7) (Tarello 2003, 2011; Mazurkevich et al. 2004; Długosz et al. 2016; Manzocchi et al. 2017; Ciuca et al. 2020; Panarese et al. 2021), epidemiological (n = 4) (Traversa et al. 2010; Giangaspero et al. 2013; Bajer et al. 2016; Genchi et al. 2019) and review studies (n = 5) (Genchi et al. 2009, 2011; Simón et al. 2009, 2012; Genchi and Kramer 2020) and clinical guidelines (n = 2) (ESDA 2017; Pennisi et al. 2020).

A total of 35 case reports of feline subcutaneous dirofilariosis in Europe have been described in Romaine (n = 1), Ukraine (n = 1), France (n = 1 but the diagnosis was made in Italy), and Italy (n = 32). Among European countries, Italy is considered the most endemic country, with the highest number of autochthonous clinical cases in cats located in northern (n = 12) and central (n = 19) and southern region (n = 1). Based on epidemiological studies performed in Italy, a total of five animals were seropositive in central Italy (Traversa et al. 2010).

## Discussion

In Europe, the first case of *D. repens* infection was described in cats in 2003 in Italy (Tarello 2003). Since then, the number of natural cases of feline subcutaneous dirofilariosis has increased. Geographic distribution of feline subcutaneous dirofilariosis follows the distribution of the infection in dogs. The prevalence of infection in endemic areas should be considered lower compared to dogs. However, very limited epidemiological information is available related to the *D. repens* infection in cats. A study performed in Poland detected a 0.7% (1/147) prevalence of *D. repens* infection by PCR test (Bajer et al. 2016). Italy is the European country with the highest number of epidemiological studies published related to *Dirofilaria* spp., including *D. repens* in dogs and cats. In this sense, an epidemiological study performed in central Italy combining different tests revealed a prevalence rate for *D. immitis* of 1.6% (5/300) and the absence of positive cats for *D. repens* (Traversa et al. 2010). More recently, a questionnaire study to know the veterinary practices performed by clinicians detected that *D. repens* is uniformly distributed along Italy, with the presence of feline cases in northern and central Italy. As a result of this study, the difficult diagnosis of *D. repens* infection in cats and dogs was also described, with the possibility that the prevalence is underestimated due to several factors, such as the presence of clinically healhty positive animals, the lack of specific serological procedures for *D. repens*, unlike heartworm disease, which counts on serological tests available to detect anti-*Dirofilaria immitis* antibodies in cats (Genchi et al. 2019).

Although the main mode of transmission of the parasite to cats is via the bite of the female mosquitoes, other non-vectorial transmission has not been described; however, transplacental transmission of microfilariae from queen to offspring has been suspected in Poland (Długosz et al. 2016). Among risk factors, lifestyle could be an epidemiologic factor, as most clinical cases reported that cats were outdoors, both owned cats and stray cats (Długosz et al. 2016; Ciuca et al. 2020).

Clinical manifestations of infection may range from absent, being infection a causal finding during elective surgery as in our case and other reports (Długosz et al. 2016), to mild and several diseases as in case of a concomitant disease (Panarese et al. 2021). In general, the most common clinical manifestations in cats are dermatological signs such as pruritus, alopecia, erythema, papular dermatitis, crusting dermatitis and less commonly the subcutaneous nodules. Other non-specific clinical signs include anorexia, lymphadenopathy, pale mucous membranes, lethargy, conjunctivitis and pain (Tarello 2011; Manzocchi et al. 2017). Although the presence of clinicopathological abnormalities is rare, laboratory findings are mild abnormalities in the count blood cell with an increase of number of eosinophils, neutrophils, and lymphocytes (Tarello 2011; Manzocchi et al. 2017; Ciuca et al. 2020), together with an increase of some biochemical parameters (Ciuca et al. 2020; Panarese et al. 2021). In our case, the laboratory findings were basophilia, hyperproteinemia due to hyperglobulinemia and polyclonal gammopathy dectected by serum protein electrophoresis. As previously described, serum amyloid A has also increased in the present report (Panarese et al. 2021).

In conclusion, the present case report confirms that cats in Spain could be infected by *D. repens* based on morphological examination and molecular result. The clinician should be aware that this nematode could be detected in cats, especially in Spain and the reaming European Mediterranean countries. In case of detection of the presence of compatible subcutaneous filarial parasites, parasite identification is necessary to discriminate between potential aberrant location of *D. immitis* parasites versus *D. repens* parasites (Supplementary Fig. 1).

### Electronic supplementary material

Below is the link to the electronic supplementary material.

Supplementary Material 1

## Data Availability

The data that support the findings of this study are available from the corresponding author upon reasonable request.
