# Supplementary figures and images for: First report on natural infection by Dirofilaria repens in a cat in Spain: case report and literature review of feline subcutaneous dirofilariosis in Europe
Source: Vet Res Commun. 2023 Nov 3;48(2):1195–201. doi: 10.1007/s11259-023-10250-7 (PMC10998798; doi:10.1007/s11259-023-10250-7)

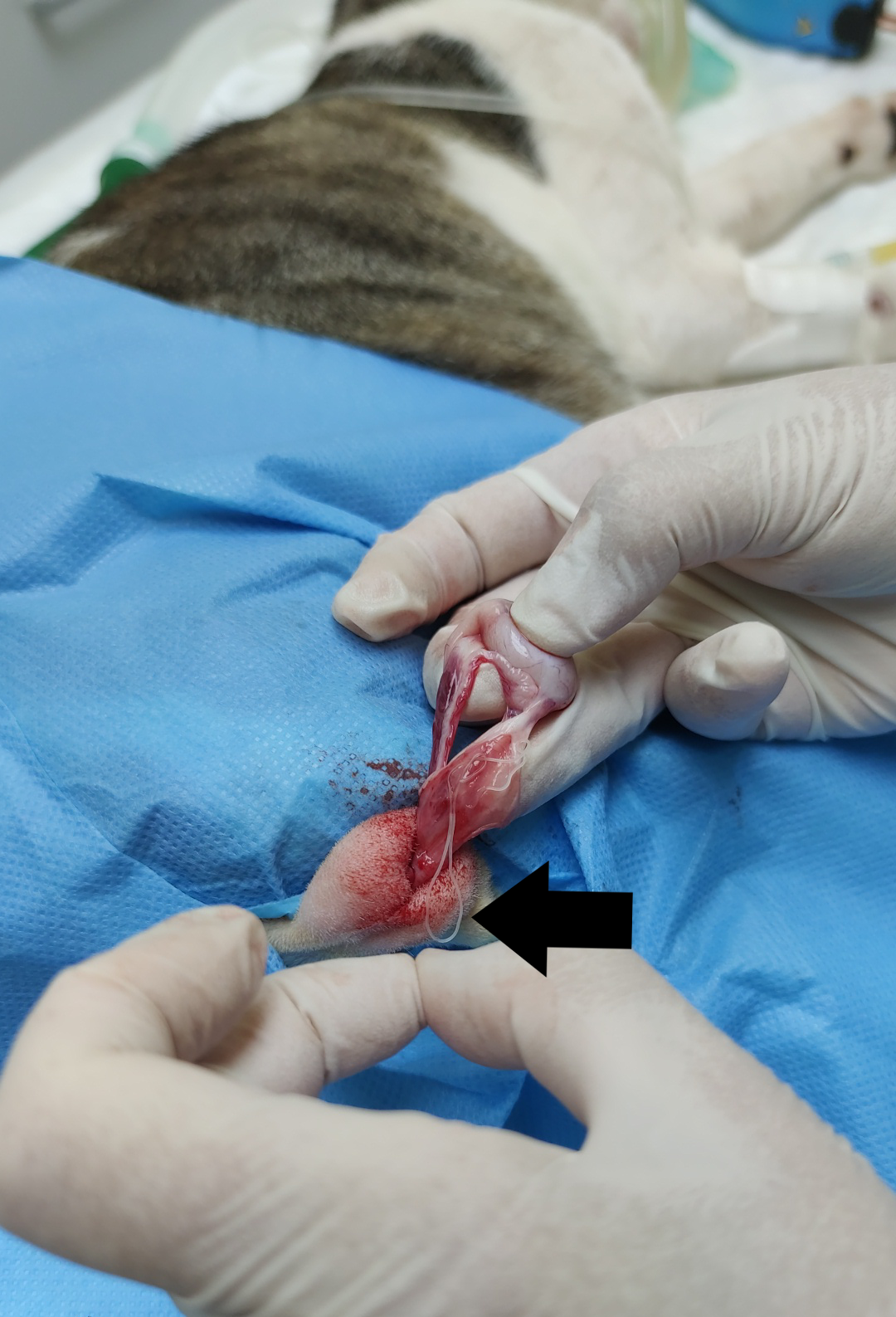

Supplement: Supplementary file 1 — Supplementary Material 1 [file 11259_2023_10250_MOESM1_ESM.tif]
